# Ageing and Health in Sub-Sahara Africa

**DOI:** 10.3389/ijph.2024.1608177

**Published:** 2025-01-03

**Authors:** Tobias Vogt, Elke Loichinger, Alyson van Raalte, Stephen Ojiambo Wandera

**Affiliations:** ^1^ Population Research Centre, Faculty of Spatial Sciences, University of Groningen, Groningen, Netherlands; ^2^ Prasanna School of Public Health, Manipal Academy for Higher Education, Manipal, India; ^3^ Global and Regional Population Dynamics Research Group, Federal Institute for Population Research (BIB), Wiesbaden, Germany; ^4^ Lifespan Inequalities Independent Research Group, Max Planck Institute for Demographic Research, Rostock, Germany; ^5^ Max Planck – University of Helsinki Center for Social Inequalities in Population Health (MaxHel Center), Rostock, Germany; ^6^ Max Planck – University of Helsinki Center for Social Inequalities in Population Health (MaxHel Center), Helsinki, Finland; ^7^ Department of Population Studies, Makerere University, Kampala, Uganda

**Keywords:** ageing, Africa, sub-Saharan Africa, multimorbidity, health

Sub-Saharan African societies have experienced substantial gains in life expectancy at birth in recent decades, with mortality reductions at all ages. These added years of life have led to an increasing number of older persons. Today, 4.8% percent of the population is older than 60 years and this share is expected to rise to 7.4% by 2050. This seemingly low share masks large absolute numbers. By 2050 158 million persons aged 60 years and older are projected to live in the region.

Despite past mortality improvements, the chances of surviving from infancy to older ages are very unevenly distributed and depend on a range of contextual factors. Girls in Botswana live 71.9 years on average, under the conditions of the 2024 life table death rates. This is a full 17 years longer than girls in Nigeria, and 5 years longer than Botswanan boys. In countries with comparatively high and increasing life expectancy such as Botswana, Rwanda, Kenya or South Africa, the number of older adults will increase steeply during the next years. These countries are also those experiencing among the lowest fertility rates in the region, a combination that makes them the forerunners of population ageing in Sub-Saharan Africa. Already today, around 5%–8% of the population in these countries is older than 60 years and this share is expected to double to 10%–16% within the next 3 decades [1].

The twelve articles in the Special Issue “*Ageing and health in Sub-Sahara Africa*” cover a wide range of topics in Cameroon, Ghana, Kenya, Nigeria, South Africa, Tanzania, Zambia, and sub-Saharan African countries overall. The described increase in the number of older adults creates challenges for policymakers and societies. Of particular concern is the vulnerability of older persons with regard to ill-health and access to health services [2]. With increasing life expectancies, older Africans are experiencing longer phases of dependency on public or private support, in combination with a rise of non-communicable diseases and disabilities [3]. Chronic conditions like obesity, diabetes or depressive symptoms are widespread. Multimorbidity is common already from middle adult ages. For example, in their study from Tanzania, Kohler et al. report that 73% of peri-urban dwellers above age 40 suffer from more than one chronic condition with women being more likely to suffer from multimorbidity than men. Mwangala et al. report that the share of frail older adults was around 13% in a Kenyan costal population and is especially high among older adults with HIV. The ageing of HIV prevalence into older age groups in combination with increasing other chronic health conditions confronts policymakers, individuals, and their families with the challenges of managing a double disease burden [3].

Increasing disease burdens and disabilities are only one dimension of older adults’ vulnerability. They are also exposed to various forms of deprivation which increases their dependency in times of need. Mobolaji shows that 75% of older Nigerians, especially older women, are multidimensionally deprived which includes lack of assets, low education and poor living standards. The importance of these individual dimensions is confirmed by a study from Zambia that emphasizes the high relevance of housing conditions and additional community level factors such as access to tap water and availability of cooking or heating fuel Banda et al.

In the absence of larger public support programs, major shares of the older Sub-Saharan African population are relying exclusively on their families to support them in times of need. This seems especially true once older adults suffer from chronic conditions and disability. However, a study from Ghana suggests that the receipt of family support is more impacted by an older adult’s ability to work rather than the prevalence of a chronic condition Hooley et al. Also family wealth as such seems to be less relevant for the risk of suffering from disability Makofane et al.

Overall, the articles in this Special Issue highlight a region coming to grips with larger populations in need of care. The articles are also informative by what they do not contain, and that is rich cross-country comparative perspectives supported by quality data. Datasets from the region remain questionably representative, and longitudinal data is scarce. Not only is this a problem for studying the determinants of healthy ageing, but it creates major limitations for monitoring the progress of Sub-Saharan countries in providing supportive environments for older populations. The challenges of individual and population ageing have been addressed in the Madrid International Plan of Action on Ageing (MIPAA). It represents a policy resource to help governments and societal stakeholder to meet these challenges. In her commentary, Schmidt discusses the emergence of MIPAA and the urgency of Sub-Saharan African countries to focus on different priority areas to prepare for population ageing which is already a reality in many of them.
